# Editorial: Screening for and Treatment of Moral Injury in Veterans/Active Duty Military With PTSD

**DOI:** 10.3389/fpsyt.2019.00596

**Published:** 2019-08-21

**Authors:** Harold G. Koenig, Donna Ames, Arndt Büssing

**Affiliations:** ^1^Department of Psychiatry and Behavioral Sciences and Department of Medicine, Duke University Medical Center, Durham, NC, United States; ^2^Department of Medicine, King Abdulaziz University, Jeddah, Saudi Arabia; ^3^School of Public Health, Ningxia Medical University, Yinchuan, China; ^4^Department of Psychiatry, VA Greater Los Angeles Healthcare System, Los Angeles, CA, United States; ^5^Department of Psychiatry, David Geffen School of Medicine at UCLA, Los Angeles, CA, United States; ^6^Quality of Life, Spirituality and Coping, Faculty of Health, Witten/Herdecke University, Witten, Germany; ^7^IUNCTUS—Competence Center for Christian Spirituality, Philosophical-Theological Academy, Münster, Germany

**Keywords:** moral injury, PTSD, definition, screening, veterans

Moral injury (MI) is a relatively new syndrome, yet one that has been around for a long time. MI often accompanies posttraumatic stress disorder (PTSD) and is especially common in active duty military (ADM) and veterans as a result of combat experiences and other military-related traumas. MI may also be common in noncombat veterans, health professionals, and even civilian populations. The purpose of this Research Topic is to define and describe MI in veterans and ADM, examine how it is assessed and differentiated from PTSD, and begin to explore ways that psychiatrists and other health professionals can identify and address it. In this issue, we present perspectives and new research on MI from around the world, including the USA and Canada, Australia, France, and Germany.

When it occurs in the military, MI has been defined as the emotional, spiritual, and moral consequences of committing and/or observing others commit transgressions of deeply held moral values during combat or combat-related circumstances (1). Another common definition describes MI as “a betrayal of what’s right, by someone who holds legitimate authority, in a high-stakes situation” (2), in other words, betrayal by commanders who may have placed service members in a position that forced them to transgress moral boundaries. Brief measures now exist that have been psychometrically validated to identify symptoms of MI among veterans and those currently in the military (3). Research has shown that >50% of ADM with PTSD symptoms have four or more symptoms of MI in the severe range (9 or 10 on a 1–10 scale) (4), and nearly 60% of veterans with PTSD have five or more such symptoms (5).

In the past decade, we have learned that moral injuries of this type can have devastating consequences on mental health, causing severe anxiety, depression, hopelessness, and suicide among ADM and veterans (6). Given the many challenges involved in successfully treating military-related PTSD, clinicians are often so focused on PTSD symptoms and comorbid disorders (mood disorders, substance abuse, risk of suicide, etc.) that they fail to recognize underlying moral injuries that may be driving these disorders (1). Growing research suggests that PTSD and MI are distinct but overlapping conditions (7). Failure to recognize and address MI may impair successful treatment of PTSD, at least partly explaining why PTSD outcomes are so poor despite the best pharmacological and psychotherapeutic treatments now available (8).

While especially common in military settings, MI is also experienced by those outside the military. Much recent attention has been paid to rising suicide rates and burnout among physicians and nurses, which may be linked to moral injuries that occur in high stakes situations involving life and death decisions that these health professionals make (9). Likewise, victims of sexual and racial abuse may experience shame, guilt, anger, and undergo spiritual struggles. Although we focus here on MI acquired in military settings, future research should seek to identify and treat noncombat veterans, civilians in high-risk professions (physicians, nurses, police, firemen, other first responders), and those with a history of trauma (abuse, rape) who may experience similar symptoms.

New approaches to the treatment of MI in the setting of PTSD are now being developed and tested in randomized controlled trials (10, 11, 12). These treatments provide hope and the promise of relief to millions of ADM and veterans who currently suffer from PTSD and related disorders. Before psychiatrists and mental health professionals can take advantage of these new treatments, however, they need to know how to identify MI, who to refer to, and what kinds of treatments are available to help those with a condition that may afflict more than half of current military personnel and veterans with PTSD symptoms. This Research Topic is designed to assist and inform in this regard.

In the first article, Koenig et al. review and discuss the definition of MI and the way that it has been conceptualized and measured among veterans and ADM, making recommendations for both investigators who conduct research in this area and clinicians who must screen for this syndrome in clinical practice. In the second article, Brémault-Phillips et al. briefly review past research on MI and mental health outcomes in the setting of PTSD among current and former military personnel. Next, Kopacz et al. illustrate this by exploring the association between loss of trust (a key symptom of MI) and mental health among 427 veterans and ADM with combat-related PTSD symptoms. Frankfurt et al. then delve into the mechanisms (direct and indirect pathways) by which MI occurs as a result of two specific types of military-related trauma in US Veterans, sexual trauma and combat exposure.

The next five articles focus on treatment. Belrose et al. present a new approach to the challenge of reintegrating soldiers with chronic PTSD back into civilian life in France. Carey and Hodgson follow with an article on how clinicians can identify and treat MI, drawing on their experience from Australia and illustrating the important role that military chaplains play in addressing this syndrome. Next, Büssing et al. draw on data from a large study of German soldiers, emphasizing the need to talk about experiences during combat, the need to forgive others, and the need to be forgiven for transgressions, ultimately leading to healing of moral injuries experienced during war. Purcell and colleagues then discuss why forgiveness is so important to US Veterans who feel guilt and shame about their actions in war, what type of forgiveness is attainable and meaningful, and what role clinicians can play in facilitating forgiveness. Finally, Smith-MacDonald et al. examine the spiritual dimensions of MI in the Canadian armed forces, describing what chaplains in this setting have to offer military personnel and their families.

This Research Topic promises to update readers on the latest research and discussions on this common, consequential, and often neglected syndrome. These articles will provide researchers with the best available tools to further explore the relationship between MI and mental health outcomes and to develop effective interventions, as well as inform and equip clinicians to identify MI in high-risk ADM and veterans and monitor response to treatment.

## Author Contributions

Each of the authors (HK, DA, and AB) have contributed intellectual content and have contributed to the actual writing of the editorial.

## Funding

Funding support for this article was provided by the Center for Aging and Human Development, Duke University Health Systems, Durham, North Carolina.

## Conflict of Interest Statement

The authors declare that the research was conducted in the absence of any commercial or financial relationships that could be construed as a potential conflict of interest.
